# Hemoptysis Due to Diffuse Alveolar Hemorrhage

**DOI:** 10.21980/J8ZP86

**Published:** 2020-07-15

**Authors:** Zoltan Buchwald, Amrita Vempati

**Affiliations:** * Creighton University School of Medicine Phoenix Program, Maricopa Medical Center, Department of Emergency Medicine, Phoenix, AZ

## Abstract

**Audience:**

This scenario was developed to educate junior and senior emergency medicine (EM) residents.

**Introduction:**

EM Model of Practice recommends that the residents are able to manage patients in a critical condition from massive hemoptysis. Mild to moderate hemoptysis can be self-limiting and often can be managed with conservative measures; however, massive hemoptysis is a life-threatening emergency that needs to be managed promptly. Mortality from massive hemoptysis is about 13%–18%.^1^ There are several causes for hemoptysis ranging from pulmonary to vascular causes. Diffuse alveolar hemorrhage (DAH) causes hemoptysis only 0.2% which makes it a very rare but devastating disease.^2^ Hemoptysis from DAH can present a significant challenge to an EM physician since it can present in various ways including chest pain, shortness of breath or hemoptysis. Up to 40% of the patients can present without hemoptysis which makes it a diagnostic dilemma.^3^ Patients presenting with massive hemoptysis from DAH require management for hypovolemic shock, hypoxic respiratory arrest and potential cardiac arrest. The physicians also need to perform adequate ventilator management to help with alveolar recruitment. This simulation case can help discuss some of the nuances of the management of hemoptysis and DAH.

**Objectives:**

By the end of this simulation session, learners will be able to: (1) recognize worsening respiratory status of a patient with hemoptysis and intervene appropriately, (2) manage a patient with severe hemoptysis and perform appropriate ventilator management, (3) manage sinus tachycardia with QT prolongation on the ECG caused by cocaine and hypomagnesemia, (4) address various etiologies of hemoptysis, (5) discuss the causes of massive hemoptysis and management options, and (6) review ventilation strategies in an intubated hypoxic patient.

**Educational Methods:**

This session was conducted using high-fidelity simulation, which was immediately followed by an in-depth debriefing session. Each session had 3 EM residents from various levels of training on the team and 7 observers. There was 1 simulation instructor running the session and 1 simulation technician who acted as a nurse.

**Research Methods:**

After the simulation and debriefing session was complete, an online survey was sent via surveymonkey.com to all the participants. The survey collected responses to the following questions: (1) the case was believable, (2) the case had right amount of complexity, (3) the case helped in improving medical knowledge and patient care, (4) the simulation environment gave me a real-life experience and, (5) the debriefing session after simulation helped improve my knowledge. A Likert scale was used to collect the responses.

**Results:**

Seven learners responded to the survey. One hundred percent of them either agreed or strongly agreed that the case was beneficial in learning and improving patient care. They also agreed that it helped in improving medical knowledge. The post-session debrief was found to be very helpful by all the learners.

**Discussion:**

High-fidelity simulation was a cost-effective yet realistic way to manage severe hemoptysis, PEA (pulseless electrical activity), and persistent hypoxia in patients with diffuse alveolar hemorrhage. Starting the case with severe hypoxia that quickly progresses to PEA helps the learner to manage the patient quickly and effectively. Overall, learners enjoyed managing the patient, followed by discussing the various management strategies.

**Topics:**

Hemoptysis, diffuse alveolar hemorrhage, medical simulation, respiratory.

## USER GUIDE

**Table t1-jetem-5-2-s1:** 

List of Resources:	
Abstract	1
User Guide	3
Instructor Materials	6
Operator Materials	17
Debriefing and Evaluation Pearls	20
Simulation Assessment	23


[Table t1-jetem-5-2-s1]

**Learner Audience:**

Emergency Medicine junior residents, senior residents

**Time Required for Implementation:**

Instructor Preparation: 20–30 minutes

Time for case: 15–20 minutes

Time for debriefing: 20–40 minutes

**Recommended Number of Learners per Instructor:**

3

**Topics:**

Hemoptysis, diffuse alveolar hemorrhage, medical simulation, respiratory.

**Objectives:**

By the end of this simulation session, the learner will be able to:

1. Recognize worsening respiratory status of a patient with hemoptysis and intervene appropriately.
2. Manage a patient with hemoptysis and perform appropriate ventilator management.
3. Manage sinus tachycardia with QT prolongation on the ECG caused by cocaine and hypomagnesemia.
4. Address various etiologies of hemoptysis.
5. Discuss the causes of massive hemoptysis and management options.
6. Review ventilation strategies in an intubated hypoxic patient.

### Linked objectives and methods

Chest pain and shortness of breath are the bread and butter of EM and the etiologies of each can vary tremendously. This case starts with the patient presenting with both chest pain and shortness of breath which allows the learners to maintain a broad differential and avoid anchoring. Learners are expected to recognize the worsening respiratory status and hypoxia and intervene in a timely fashion (Objective #1). Learners are further challenged by the patient going into PEA arrest requiring them to follow ACLS (advanced cardiac life support) protocol. After return of spontaneous circulation (ROSC), patient will continue to be hypoxic due to worsening alveolar hemorrhage, and the learners will need to perform appropriate ventilator management (Objective #2). In a patient with cocaine abuse, ECG changes and electrolyte abnormalities are common. This case requires learners to recognize and manage sinus tachycardia with QT prolongation on ECG caused by cocaine and hypomagnesemia (Objective #3). In addition, learners are expected to administer antibiotics and steroids to cover for the etiologies of patient’s respiratory status. Patients with severe hemoptysis need a bronchoscopy to stop the bleeding. Thus, the learners will need to consult a critical care pulmonologist/intensivist from bronchoscopy and to disposition (Objective #4). During the debriefing session, the learners will be expected to discuss the wide range etiologies of massive hemoptysis and management options (Objective #5). They will also need to discuss how they managed the ventilator during the case, or what they could have done to improve patient’s hypoxia (Objective #6).

### Recommended pre-reading for instructor

- Shah S, Cioe E, Endrizzi J. Diffuse alveolar hemorrhage in the ED: pearls & pitfalls. emDocs. http://www.emdocs.net/diffuse-alveolar-hemorrhage-in-the-ed-pearls-pitfalls/. Published June 4, 2016. Accessed April 21, 2019.
- Nickson C. Diffuse alveolar haemorrhage. Life in the Fast Lane. https://litfl.com/diffuse-alveolar-haemorrhage/. Updated April 9, 2019. Accessed April 21, 2019.
- Park MS. Diffuse alveolar hemorrhage. *Tuberc Respir Dis (Seoul)*. 2013;74(4):151–162. doi:10.4046/trd.2013.74.4.151.
- Kusunoki M, Umegaki T, Shoji T, et al. Severe progressive diffuse alveolar hemorrhage in a patient with systemic lupus erythematosus. *Case Reports in Critical Care*. 2018. doi:10.1155/2018/9790459.
- Farkas J. PulmCrit-APRV: Resurrection of the open-lung strategy? https://emcrit.org/pulmcrit/aprv/. Published November 20, 2017. Accessed April 21, 2019.
- Davidson K, Shojaee S. Managing Massive Hemoptysis. *Chest*. 2020;157(1):77–88. doi:10.1016/j.chest.2019.07.012.

### Results and tips for successful implementation

This session was conducted on a total of 9 EM residents in various levels of training, and 21 EM residents served as observers. One actor served as a nurse. Allowing the team to assign roles prior to starting the case helped in running the case smoothly.

Depending on the level of training, the case may need to be started at a lower oxygen (O_2_) saturation and higher respiratory rate. The patient’s breathing noises can be made by the instructor to give the learners a real-life experience. When the patient is being switched over from a nasal cannula to non-rebreather mask, the O_2_ saturation will rapidly start going down to allow for PEA arrest and also to prompt the learners to intubate. After ROSC, O_2_ saturation will need to stay low to prompt the learners to do ventilator management. Novice learners may need prompting to manage post-intubation persistent hypoxia, to obtain CT of the chest, and to administer steroids and antibiotics.

After the simulation and debriefing session were complete, an online survey was sent via surveymonkey.com to all the 9 participants. The responses were collected on a Likert scale of 1 to 5 with 1 being “Strongly disagree” and 5 being “Strongly agree.” The survey collected responses to the following questions:

1. The case was believable.
2. The case had right amount of complexity.
3. The case helped in improving medical knowledge and patient care.
4. The simulation environment gave me a real-life experience.
5. The debriefing session after simulation helped improve my knowledge.

A total of 7 responses were received. Below is a chart of the responses we received.[Fig f1-jetem-5-2-s1]

**Figure f1-jetem-5-2-s1:**
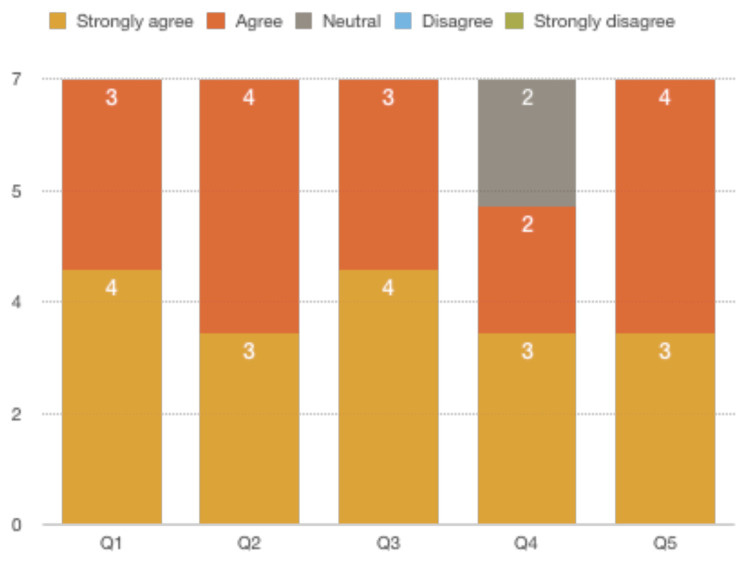


All of the respondents either agreed or strongly agreed that the case was beneficial in learning and in improving medical knowledge and patient care. They also agreed that it had the right amount of complexity. Two respondents were neutral while 5 agreed or strongly agreed that the case gave them a real-life experience. The post-session debrief was found to be helpful by all the respondents.

## Supplementary Information































## Figures and Tables

**Table t2-jetem-5-2-s1:** 

#1	Electrocardiogram (ECG) - sinus tachycardia with QT prolongation
#2	Complete blood count (CBC)
#3	Basic metabolic panel (BMP)
#4	Lactic acid level
#5	Troponin
#6	Urinalysis (UA)
#7	Urine drug screen (UDS)
#8	Direct laryngoscopy view
#9	Initial chest radiograph (CXR)
#10	Post-intubation CXR
#11	Computed Tomography (CT) scan of the chest

**Table t3-jetem-5-2-s1:** 

**Vitals:**	Heart Rate (HR) 110	Blood Pressure (BP) 190/100	Respiratory Rate (RR) 25
	Temperature (T) 36.7°C	Oxygen Saturation (O_2_Sat) 89% on nasal cannula	

**Figure f2-jetem-5-2-s1:**
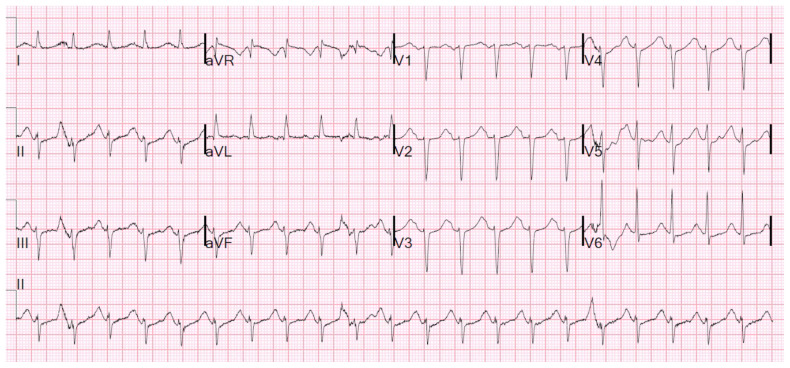


**Table t4-jetem-5-2-s1:** 

*Complete blood count (CBC)*	
White blood count (WBC)	25.0 ×1000/mm^3^
Hemoglobin (Hgb)	10.0 g/dL
Hematocrit (HCT)	27.0%
Platelet (Plt)	210 x1000/mm^3^

**Table t5-jetem-5-2-s1:** 

*Basic metabolic panel (BMP)*	
Sodium	129 mEq/L
Chloride	79 mEq/L
Potassium	3.0 mEq/L
Bicarbonate (HCO_3_)	20 mEq/L
Blood Urea Nitrogen (BUN)	20 mg/dL
Creatine (Cr)	1.2 mg/dL
Glucose	120 mg/dL
Calcium	9.0 mg/dL

**Table t6-jetem-5-2-s1:** 

*Lactic Acid*	3.2 mmol/L

**Table t7-jetem-5-2-s1:** 

*Magnesium*	0.5 mEq/L

**Table t8-jetem-5-2-s1:** 

*Troponin*	<0.05 ng/L

**Table t9-jetem-5-2-s1:** 

*Urinalysis (UA)*	
Color	yellow
Appearance	clear
Specific gravity	1.10
pH	7.0
Glucose	negative
Bilirubin	negative
Ketones	1+
Protein	2+
Leukocyte esterase	negative
Nitrites	negative
White blood cells (WBC)	0–5 WBCs/high powered field (HPF)
Red blood cells (RBC)	0–5 RBCs/HPF
Squamous epithelial cells	0–5 cells/HPF

**Table t10-jetem-5-2-s1:** 

*Urine drug screen*	
Amphetamine	negative
Barbiturate	negative
Benzodiazepine	negative
Cocaine	positive
Marijuana	positive
Methadone	negative
Methamphetamine	negative
Opiate	negative
Phencyclidine	negative
Tricyclic antidepressants	negative

**Figure f3-jetem-5-2-s1:**
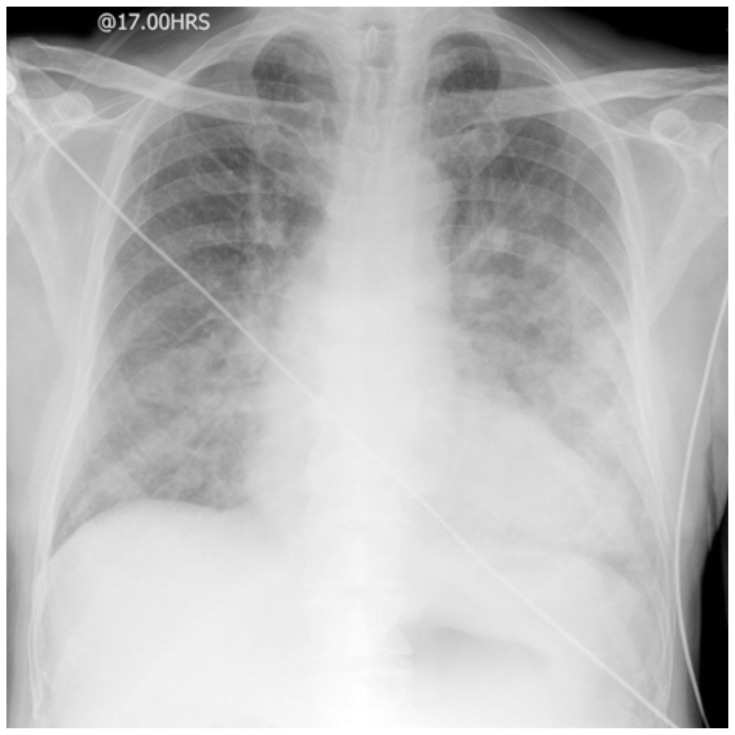


**Figure f4-jetem-5-2-s1:**
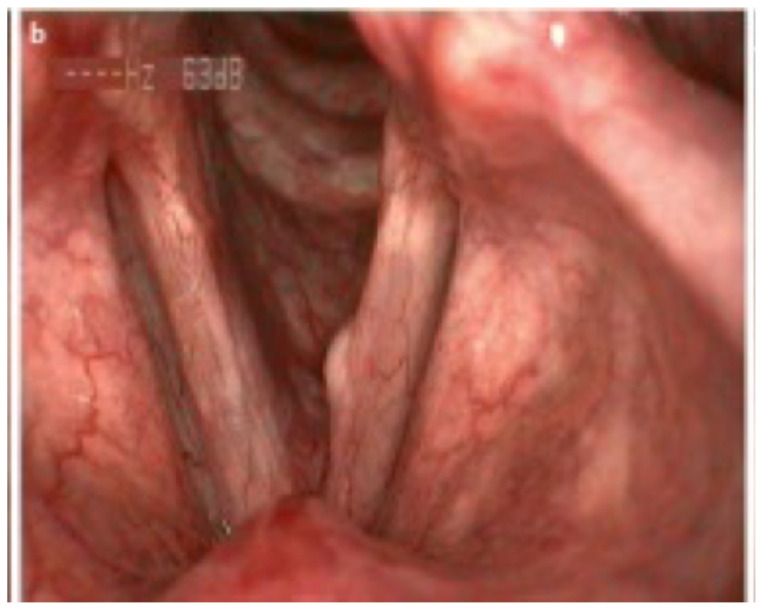


**Figure f5-jetem-5-2-s1:**
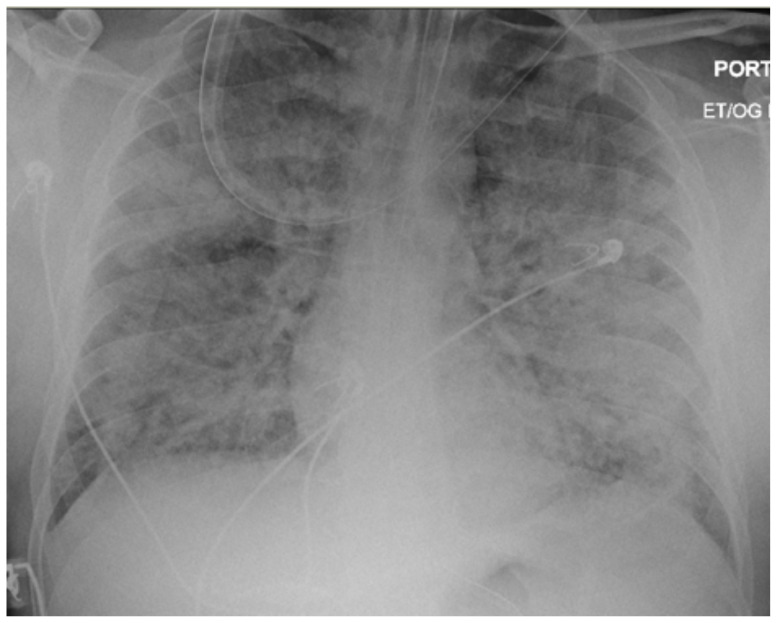


**Figure f6-jetem-5-2-s1:**
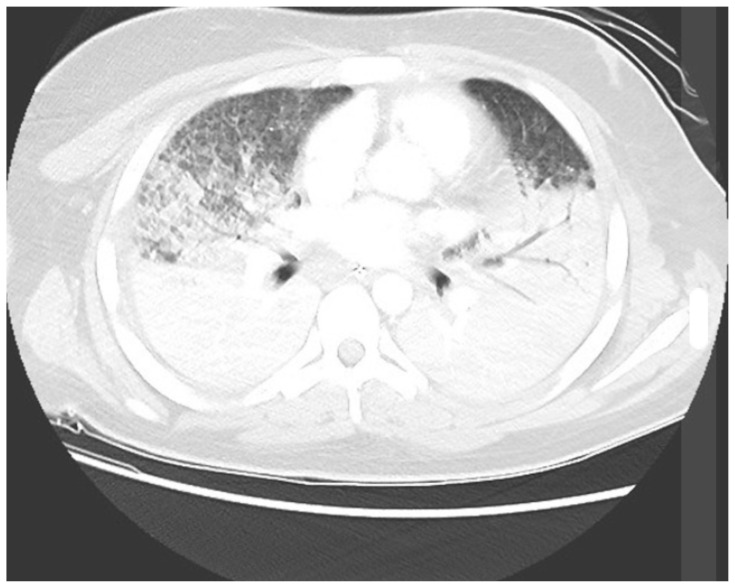


**Table t11-jetem-5-2-s1:** SIMULATION EVENTS TABLE:

Minute (state)	Participant action/trigger	Patient status (simulator response) & operator prompts	Monitor display (vital signs)
0:00 (Baseline)	EMS report Team obtains the history and performs a physical exam	Patient only speaks in a few words and provides history. Patient continues to get more tachypneic. He has hemoptysis in the ED. Patient continues to say “I… can’t …breathe…”	BP 190/100 HR 110 RR 25 T 36.7 O_2_sat 81% on NC
2:00–4:00	Team may request for ECG Order more lab studies CXR Ordered	If ECG is not ordered, nurse may prompt, “Why is he having shortness of breath? Is there something we can check?” or “Should I get an ECG?” If ECG is ordered, nurse may ask, “Is the ECG normal? Why is he feeling this way?” ECG will be provided. Laboratory studies will not be given at this time. If CXR is ordered, ask if team wants the patient to leave the department for it. Patient continues to get more short of breath. He coughs up bright red blood.	BP 195/100 HR 110 RR 32 T 36.7 O_2_sat 76% on NC
4:00–7:00	Assess airway and respiratory status Request to switch to NIPPV/intubate	If team attempts NRB or NIPPV Patients says “NOO!! I… don’t…want…anything… on… my face!!” If attempts at escalating to NIPPV/intubation are not made, make the patient more tachypneic and noises of respiratory distress. Nurse may also prompt asking, “Is there something more we can do to help him?” Patient will refuse any attempts at mask placement. He will continue to get more hypoxic, and then go into PEA arrest. If the learners attempt to intubate prior to patient going into PEA, the patient will progress to PEA while intubation is happening. If the learners attempt to intubate, the patient will quickly desaturate into the 50’s and progress into PEA arrest.	BP 194/99 HR 115 RR 30 T 36.7 O_2_sat 69% on NC Transition to PEA
7:00–8:00	Recognize PEA	PEA will occur as the patient becomes more hypoxic and attempts at intubation are made. If PEA is not recognized, nurse may prompt saying, “I don’t feel a pulse” or “He is not responding.”	PEA
8:00–10:00	Perform CPR Administer Epinephrine Intubation	PEA on the monitor. Patient can be bagged but will be hypoxic requiring intubation. No ROSC until intubation is performed. If intubation is performed, proceed to Status B. If not, proceed to Status A.	PEA arrest
10:00–13:00 (Status A)	Perform CPR Administer Epinephrine Intubation	If intubation or adequate bagging is not performed, PEA will continue. Nurse may prompt “What can we do to help with PEA? Do you think it’s because he could not breath?” to allow learners to move to intubation. Move to Status B once the learners take the prompt and secure an airway.	PEA arrest
10:00–13:00 (Status B)	Request to be placed on ventilator after patient receives ROSC May request consultant to be called	Patient intubated. ROSC will be obtained. Hypoxia in the 80’s will continue. Consultants will not be available. Nurse will ask “What ventilator settings do you want?”	T 37.0 BP 150/80 HR 100 89% on ventilator
13:00–14:00	Recognize hypoxia Ventilator management	Patient will continue to be hypoxic after ROSC and on ventilator. If hypoxia is not recognized, nurse can prompt, “Why is his O2 saturation still low?” If learners are struggling with ventilator management, consultants/RT may be helpful with suggesting management options.	T 37.0 BP 160/85 HR 100 O_2_sat 87% on ventilator Hypoxia may improve to 90’s.
14:00–16:00	Order CT chest Determine if stable for a CT	Pulmonologist/intensivist will call back after ventilator management happens and may prompt regarding obtaining CT and steroid administration. Nurse will be concerned about sending the patient to CT scan with hypoxia. Ask if the team wants the patient to go to the CT chest now.	T 37.0 BP 165/85 HR 100 O_2_sat 92% on ventilator
16:00–20:00	IV antibiotic and steroid administration Correct electrolytes	If electrolytes are not corrected, consultants will prompt, “Is the ECG normal?” If CT was not ordered, consultant may request a CT. CT results given.	T 37.0 BP 170/90 HR 96 O_2_sat 92% on ventilator
Case completion	Admit to the ICU	Consultant will suggest admission to the ICU.	

**Table t12-jetem-5-2-s1:** 

**Critical Actions:** Assess airway, breathing and circulation Obtain a thorough history and perform a complete physical examination Manage respiratory distress and worsening hypoxia with intubation Recognize the sinus tachycardia and QT prolongation on ECG caused by cocaine abuse and hypomagnesemia and manage it appropriately Follow ACLS protocol for pulseless electrical activity Perform recruitment maneuvers and ventilator management for persistent hypoxia Obtain appropriate laboratory studies and a CT of the chest Administer antibiotics and steroids Consult pulmonologist/intensivist for bronchoscopy and disposition Admit to the intensive care unit	0:00

**Table t13-jetem-5-2-s1:** 

	Milestone	Did not achieve level 1	Level 1	Level 2	Level 3
1	**Emergency Stabilization (PC1)**	□ Did not achieve Level 1	□ Recognizes abnormal vital signs	□ Recognizes an unstable patient, requiring intervention Performs primary assessment Discerns data to formulate a diagnostic impression/plan	□ Manages and prioritizes critical actions in a critically ill patient Reassesses after implementing a stabilizing intervention
2	**Performance of focused history and physical (PC2)**	□ Did not achieve Level 1	□ Performs a reliable, comprehensive history and physical exam	□ Performs and communicates a focused history and physical exam based on chief complaint and urgent issues	□ Prioritizes essential components of history and physical exam given dynamic circumstances
3	**Diagnostic studies (PC3)**	□ Did not achieve Level 1	□ Determines the necessity of diagnostic studies	□ Orders appropriate diagnostic studies. Performs appropriate bedside diagnostic studies/procedures	□ Prioritizes essential testing Interprets results of diagnostic studies Reviews risks, benefits, contraindications, and alternatives to a diagnostic study or procedure
4	**Diagnosis (PC4)**	□ Did not achieve Level 1	□ Considers a list of potential diagnoses	□ Considers an appropriate list of potential diagnosis May or may not make correct diagnosis	□ Makes the appropriate diagnosis Considers other potential diagnoses, avoiding premature closure
5	**Pharmacotherapy (PC5)**	□ Did not achieve Level 1	□ Asks patient for drug allergies	□ Selects an medication for therapeutic intervention, consider potential adverse effects	□ Selects the most appropriate medication and understands mechanism of action, effect, and potential side effects Considers and recognizes drug-drug interactions
6	**Observation and reassessment (PC6)**	□ Did not achieve Level 1	□ Reevaluates patient at least one time during case	□ Reevaluates patient after most therapeutic interventions	□ Consistently evaluates the effectiveness of therapies at appropriate intervals
7	**Disposition (PC7)**	□ Did not achieve Level 1	□ Appropriately selects whether to admit or discharge the patient	□ Appropriately selects whether to admit or discharge Involves the expertise of some of the appropriate specialists	□ Educates the patient appropriately about their disposition Assigns patient to an appropriate level of care (ICU/Tele/Floor) Involves expertise of all appropriate specialists
9	**General Approach to Procedures (PC9)**	□ Did not achieve Level 1	□ Identifies pertinent anatomy and physiology for a procedure Uses appropriate Universal Precautions	□ Obtains informed consent Knows indications, contraindications, anatomic landmarks, equipment, anesthetic and procedural technique, and potential complications for common ED procedures	□ Determines a back-up strategy if initial attempts are unsuccessful Correctly interprets results of diagnostic procedure
20	**Professional Values (PROF1)**	□ Did not achieve Level 1	□ Demonstrates caring, honest behavior	□ Exhibits compassion, respect, sensitivity and responsiveness	□ Develops alternative care plans when patients’ personal beliefs and decisions preclude standard care
22	**Patient centered communication (ICS1)**	□ Did not achieve level 1	□ Establishes rapport and demonstrates empathy to patient (and family) Listens effectively	□ Elicits patient’s reason for seeking health care	□ Manages patient expectations in a manner that minimizes potential for stress, conflict, and misunderstanding. Effectively communicates with vulnerable populations, (at risk patients and families)
23	**Team management (ICS2)**	□ Did not achieve level 1	□ Recognizes other members of the patient care team during case (nurse, techs)	□ Communicates pertinent information to other healthcare colleagues	□ Communicates a clear, succinct, and appropriate handoff with specialists and other colleagues Communicates effectively with ancillary staff

## INSTRUCTOR MATERIALS

**Case Title:** Hemoptysis due to Diffuse Alveolar Hemorrhage

**Case Description & Diagnosis (short synopsis):** A 35-year-old male brought in by emergency medical services (EMS) to the emergency department (ED) for sudden onset chest pain and shortness of breath. He initially complains of coughing up only a spoonful of blood. Upon further questioning, he states he had done some crack cocaine after which all of these symptoms had started. He also admits that he has been coughing up blood for several months now. The patient is initially tachypneic and hypoxic and was placed on nasal cannula by EMS. Upon arrival to the ED, he speaks in a few words and is visibly short of breath. He is coughing up blood clots while getting more hypoxic. He refuses placement of non-rebreather or noninvasive positive pressure ventilation (NIPPV). As the hypoxia worsens, the patient will go into pulseless electrical activity. After intubation and ROSC, patient will continue to be hypoxic due to worsening hemorrhage requiring recruitment maneuvers and ventilator management.

**Equipment or Props Needed:**

High-fidelity simulator

Basin with red colored gel for hemoptysis

Airway supplies:

- Nasal cannula
- Non-rebreather mask
- Laryngoscope and blades
- Endotracheal tube and stylet
- Bag-valve mask

IV supplies:

- 2 18g angiocatheters
- IV tubing
- Saline

Medications:

- Etomidate
- Succinylcholine
- Albuterol
- Methylprednisolone
- Epinephrine
- Sodium bicarbonate
- Nicardipine
- Azithromycin
- Ceftriaxone

Cardiac monitor

Gloves

**Confederates needed:**

Operator may play nurse or EMS

**Stimulus Inventory:** [Table t2-jetem-5-2-s1]

**Background and brief information:** A 35-year-old male brought in by EMS to tertiary care center for sudden onset chest pain and shortness of breath.

**Initial presentation:** The patient is brought in by EMS and he complains of shortness of breath. He starts coughing up blood clots upon arrival. In addition, he has tachypnea, tachycardia, and is hypertensive.

**How the scene unfolds:** Upon arrival to the ED, the learners should start by assessing the patient’s respiratory status and airway. They may choose to switch from nasal cannula to non-rebreather and may discuss placing the patient on NIPPV. However, patient will continue to get more short of breath, hypoxic and confused, and refuses any masks on the face. The learners will have to discuss intubation when the patient inevitably goes into PEA as hypoxia worsens. The learners will need to perform cardiopulmonary resuscitation (CPR), administer epinephrine and prepare to intubate the patient. After intubation and bagging the patient, ROSC will occur; however, patient will continue to be hypoxic in the low 80’s. The team will have to do alveolar recruitment maneuvers to improve the hypoxia. Once the hypoxia improves, the team will have to obtain a CT of the chest to diagnose the cause of hemoptysis. They will also need to consult pulmonologist/intensivist for bronchoscopy and to disposition the patient. In addition, they need to administer IV antibiotics and IV steroids. The case will conclude when they send the patient to the intensive care unit.

**Critical actions:**

1. Assess airway, breathing and circulation
2. Obtain a thorough history and perform a complete physical examination
3. Manage respiratory distress and worsening hypoxia with intubation
4. Recognize the sinus tachycardia and QT prolongation on ECG caused by cocaine abuse and hypomagnesemia and manage it appropriately
5. Follow ACLS protocol for pulseless electrical activity
6. Perform recruitment maneuvers and ventilator management for persistent hypoxia
7. Obtain appropriate laboratory studies and a CT of the chest
8. Administer antibiotics and steroids
9. Consult pulmonologist/intensivist for bronchoscopy and disposition
10. Admit to the intensive care unit

**Case Title:** Hemoptysis due to Diffuse Alveolar Hemorrhage

**Chief Complaint:** Chest pain, shortness of breath, and “coughing up blood”[Table t3-jetem-5-2-s1]

**General Appearance:** Diaphoretic, coughing and tachypneic with obvious

**Primary Survey:**

- **Airway:** speaking in words
- **Breathing:** diffuse rales with tachypnea and hemoptysis
- **Circulation:** tachycardic with bilateral radial and dorsal pedis pulses

**History:**

- **History of present illness:** EMS reports that they got called for sudden onset chest pain. He was getting more short of breath and started coughing up blood on the way to the hospital. They obtained a 12-lead ECG.
  Patient is able to provide history in 1-word sentences. Chest pain is sharp, middle of the chest. Pain worsens with breathing. He also complains of shortness of breath which started after the chest pain. He also coughed up some blood in the sputum on the way to the ED. The sputum was completely bloody when he coughed it up today. He denies any fever, chills, abdominal pain, nausea, vomiting, headache, or chills. He also denies any trauma to the chest.
  If asked, patient reports that he had snorted crack cocaine and he started experiencing chest pain shortly after. He has been having cough with bloody sputum for some time now. He has not seen a physician in a long time.
- **Past medical history:** Hypertension
- **Past surgical history:** Exploratory laparotomy from a gunshot wound about 10 years ago
- **Medications:** none
- **Allergies:** none
- **Social history:** crack cocaine
- **Family history:** unknown

**Secondary Survey/Physical Examination:**

- **General appearance:** diaphoretic, coughing and tachypneic with obvious respiratory distress
- **HEENT:**
  ○ **Head:** diaphoresis on the forehead
  ○ **Eyes:** pupils are 4mm, round and reflective to light
  ○ **Ears:** normal tympanic membranes bilaterally
  ○ **Throat:** dry mucosal membranes, no visible blood in the oropharynx
- **Neck:** no thyromegaly
- **Heart:** S1 and S2 normal but tachycardic. No murmurs or gallops
- **Lungs:** rales in the bilateral upper and lower lobes without wheezing
- **Abdominal/GI:** Midline well-healed exploratory laparotomy scar. Soft, non-tender, non-distended. No rigidity or voluntary guarding. No rebound tenderness.
- **Genitourinary:** within normal limits
- **Rectal:** Negative
- **Extremities:** within normal limits
- **Back:** within normal limits
- **Neuro:** follows commands, CN II-XII intact. No focal deficits.
- **Skin:** diaphoretic, no rashes
- **Lymph:** within normal limits
- **Psych:** within normal limits

**Results:**

*Electrocardiogram (EKG)*

Sinus Tachycardia with QT prolongation

Smith S. Alcohol withdrawal and sinus tachycardia. Dr. Smith’s ECG Blog. http://hqmeded-ecg.blogspot.com/2018/03/alcohol-withdrawal-and-sinus-tachycardia.html. March 8, 2018. Accessed January 12, 2020. CC BY-NC 4.0.[Fig f2-jetem-5-2-s1]

[Table t4-jetem-5-2-s1] [Table t5-jetem-5-2-s1] [Table t6-jetem-5-2-s1] [Table t7-jetem-5-2-s1] [Table t8-jetem-5-2-s1] [Table t9-jetem-5-2-s1] [Table t10-jetem-5-2-s1] *Chest Radiograph (CXR)*

Gaillard F, Jones J. AP portable CXR of a patient in acute pulmonary oedema. Wikimedia Commons. https://commons.wikimedia.org/wiki/File:AP_portable_CXR_of_a_patient_in_acute_pulmonary_oedema.jpg. July 23, 2009. Accessed Jan 12, 2020. CC BY-SA 3.0.[Fig f3-jetem-5-2-s1]

*Direct Laryngoscopy*

Minimal blood and redness in the vocal cords after suctioning

Image source: Bohlender J. Vocal folds. Wikimedia Commons. https://commons.wikimedia.org/wiki/File:Vocal_Folds.jpg. November 10, 2016. Accessed January 12, 2020. CC BY 3.0.[Fig f4-jetem-5-2-s1]

*Post-intubation Chest Radiograph (CXR#2)*

Image source: Heilman J. Severe ARDS. Person is intubated with an OG in place. Wikimedia Commons. https://commons.wikimedia.org/wiki/File:ARDSSevere.png. September 17, 2017. Accessed January 12, 2020. CC BY-SA 4.0.[Fig f5-jetem-5-2-s1]

*Computed tomography (CT) scan of the chest – post intubation*

CT read: Ground-glass opacities bilaterally with consolidations. Differential includes infectious, inflammatory vs hemorrhage. Correlate clinically.

Image source: Muzio BD. Diffuse alveolar hemorrhage. Radiopaedia: rID 61650. https://radiopaedia.org/articles/diffuse-alveolar-haemorrhage?lang=us. CC BY-NC-SA 3.0.[Fig f6-jetem-5-2-s1]

## OPERATOR MATERIALS

### Diagnosis

[Table t11-jetem-5-2-s1]Hemoptysis secondary to diffuse alveolar hemorrhage

### Disposition

Admission to the ICU

## DEBRIEFING AND EVALUATION PEARLS

### Hemoptysis Due to Diffuse Alveolar Hemorrhage

#### Hemoptysis

Massive hemoptysis is quantified as 100–600mL of blood in a 24-hour period. Causes of hemoptysis can be memorized with mnemonic BATTLE CAMP: Bronchitis, Bronchiectasis, Aspergilloma, Tumor, Tuberculosis, Lung abscess, Emboli (pulmonary), Coagulopathy, Autoimmune disorders, AV (arteriovenous) malformation, Alveolar hemorrhage, Mitral stenosis, and Pneumonia.^4^

In the ED, workup should include ruling out emergent causes of the hemoptysis. Therefore, CBC with differential, metabolic panel, blood cultures, blood gases, erythrocyte sedimentation rate, C-reactive protein, urinalysis, urine drug screen and CXR aid in diagnosis of the underlying cause and complications.^5^ CT scan of the chest with IV contrast can help in determining the extent of the lung involvement and in ruling out infection, tumor, or pulmonary embolism. CXR may show diffuse infiltrative opacification with predilection towards the mid-zones. CT scan may show ground-glass opacities or consolidation.^6^ If the patient has underlying autoimmune disease or associated pulmonary or renal disease, specific antibodies for these diseases may be ordered.

Management in the ED includes focusing on airway, breathing and circulation (ABCs). Patients with massive hemoptysis will require immediate endotracheal (ET) intubation. CXR can be helpful in determining which lung may be bleeding more. Airway management strategies for massive hemoptysis are discussed in a separate section below. In addition to airway management and ventilation, patients may require intravenous fluid and packed red blood cell administration. The underlying causes of hemoptysis need to be addressed including steroid or antibiotic administration, coagulopathy reversal or anticoagulation. After resuscitation, bronchoscopy can be both diagnostic and therapeutic.^7^,^8^

#### Diffuse Alveolar Hemorrhage (DAH)

DAH occurs in the pulmonary microcirculation; it is specifically characterized by bleeding into the alveoli. There are a variety of disorders associated with DAH; however, it is marked by 3 distinct pathologies of the lung: 1) Pulmonary capillaritis, 2) Bland pulmonary hemorrhage and 3) Diffuse alveolar damage.^9^

Pulmonary capillaritis is characterized by neutrophilic infiltration, fibrinoid necrosis of alveolar and capillary walls, and anti-glomerular basement membrane disease. Pathologic capillaritis is due to various autoimmune conditions including Wegener’s granulomatosis, Churg-Strauss syndrome, microscopicpolyangitis, systemic lupus erythematosus, rheumatoid arthritis, etc.

Bland pulmonary hemorrhage is defined by red blood cells leakage into the alveoli without any inflammation or damage of the alveolar structures. Bland hemorrhage is generally associated with bleeding disorders, anticoagulant therapies, elevated end diastolic pressures, and can occasionally be caused by Goodpasture’s syndrome and inhalation drugs such as crack cocaine.

Diffuse alveolar damage causes the formation of alveolar edema and intra-alveolar hyaline membrane. Diffuse alveolar damage is the underlying pathology associated with acute respiratory distress syndrome (ARDS). A variant of ARDS caused by crack cocaine is often termed “crack lung.” 10, 11

Clinical presentation can include hemoptysis, anemia, and respiratory failure. However, up to a third of patients present without any hemoptysis due to the resorptive capacity of the lungs. Some of the other non-specific symptoms include cough, dyspnea, chest pain, and fever. 10 Frequent chest radiograph findings include diffuse or patchy opacities. Laboratory studies are non-specific but may help with making the diagnosis. These laboratory studies include a complete blood count with evidence of anemia, an arterial blood gas which can demonstrate the level of hypoxia, and other studies indicating coagulative dysfunction such as prothrombin time. Specific antibodies such as anti-nuclear antibody (ANA), cytoplasmic or peripheral antineutrophil cytoplasmic antibodies (C-ANCA or P-ANCA) can be considered to guide further testing.^11^

Treatment of DAH is symptomatic and primarily focuses on addressing ABCs as discussed above. In a patient with known history of underlying autoimmune disorders, methylprednisolone 500–2000mg and cyclophosphamide 2mg/kg can be administered. Intubation and ventilation is frequently required in DAH.^10^ However, ventilation may prove difficult in patients with severe alveolar hemorrhage and associated ARDS.^12^ Recruitment maneuvers such as ARDSnet low tidal-volume ventilation and Airway Pressure Release Ventilation (APRV) may be attempted.^12^,^13^ ARDSnet focuses on high positive end expiratory pressures in order to recruit and maintain open alveoli whereas APRV is a less commonly known inverse ventilation method that gives a high level of continuous pressure and intermittently releases the pressure to allow expiration.^13^,^14^

#### Airway Management

Airway management in massive hemoptysis can be very daunting for an EM physician. Patients are very good at coughing out the blood from the airway. Therefore, ET intubation should be done in the following circumstances: ineffective cough, worsening respiratory failure, and/or to obtain imaging studies or procedures.^15^ There are a few strategies that can improve success in securing an airway. Raise the head of the bed to lift the epiglottis out of the pool of blood. During intubation, suction assisted laryngoscopy and airway decontamination (SALAD) technique can be used to suction out clots and blood. During intubation, the ET tube can be turned 90 degrees toward the side to perform mainstem bronchus intubation. In addition, a bougie can be very helpful in intubating a bronchus. Larger ET tubes (size 8) are typically recommended to allow for eventual bronchoscopy.^7^ Another method is using a smaller size tube (size 7) to intubate the right main stem bronchus and if that turns out to be the bleeding side, then adding another tube to intubate the second main stem bronchus.^16^ This technique may require a lot of skill and patience to establish a successful airway. After intubation, patient can be placed in lateral decubitus with the bleeding lung down.^7^ If the bleeding is bilateral, placing the patient in a Trendelenburg position can help reduce retention of the hemorrhage in the lungs, thus improving ventilation.

#### Other Debriefing Points

How was communication between team members?

What was the initial differential? How did it develop?

## SIMULATION ASSESSMENT

*Hemoptysis Due to Diffuse Alveolar Hemorrhage*

Learner: _________________________________________

***Assessment Timeline***

This timeline is to help observers assess their learners. It allows observer to make notes on when learners performed various tasks, which can help guide debriefing discussion.[Table t12-jetem-5-2-s1]

**Critical Actions:**

□ Assess airway, breathing and circulation
□ Obtain a thorough history and perform a complete physical examination
□ Manage respiratory distress and worsening hypoxia with intubation
□ Recognize the sinus tachycardia and QT prolongation on ECG caused by cocaine abuse and hypomagnesemia and manage it appropriately
□ Follow ACLS protocol for pulseless electrical activity
□ Perform recruitment maneuvers and ventilator management for persistent hypoxia
□ Obtain appropriate laboratory studies and a CT of the chest
□ Administer antibiotics and steroids
□ Consult pulmonologist/intensivist for bronchoscopy and disposition
□ Admit to the intensive care unit

**Summative and formative comments:**

**Milestones assessment:** [Table t13-jetem-5-2-s1]
