# Supplementary figures and images for: Hemoptysis Due to Diffuse Alveolar Hemorrhage
Source: J Educ Teach Emerg Med. 2020 Jul 15;5(3):S1–S27. doi: 10.21980/J8ZP86 (PMC10332551; doi:10.21980/J8ZP86)

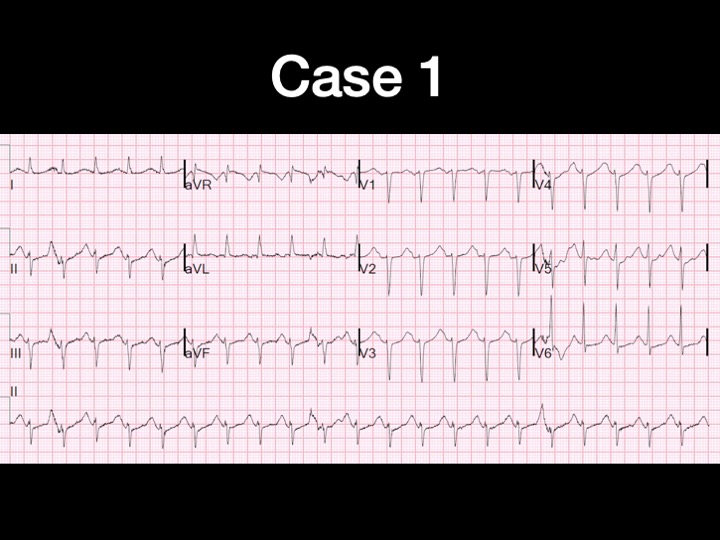

Supplement: Supplementary file 1 [file jetem-5-2-s1-supp.jpg]

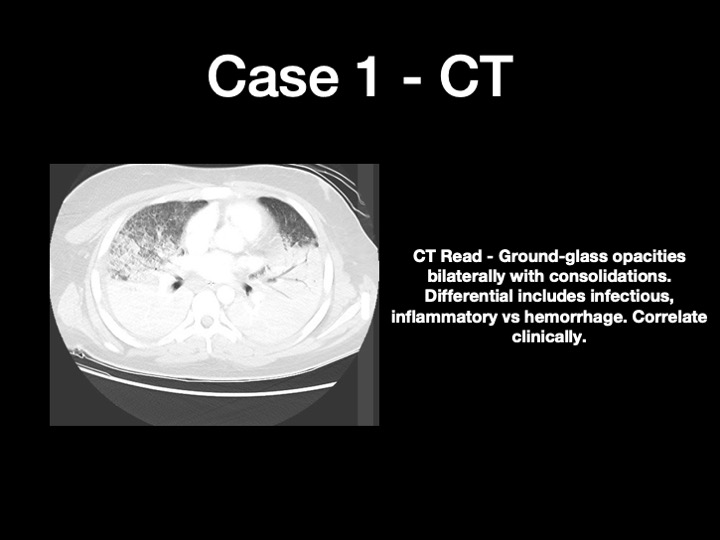

Supplement: Supplementary file 2 [file jetem-5-2-s1-supp1.jpg]

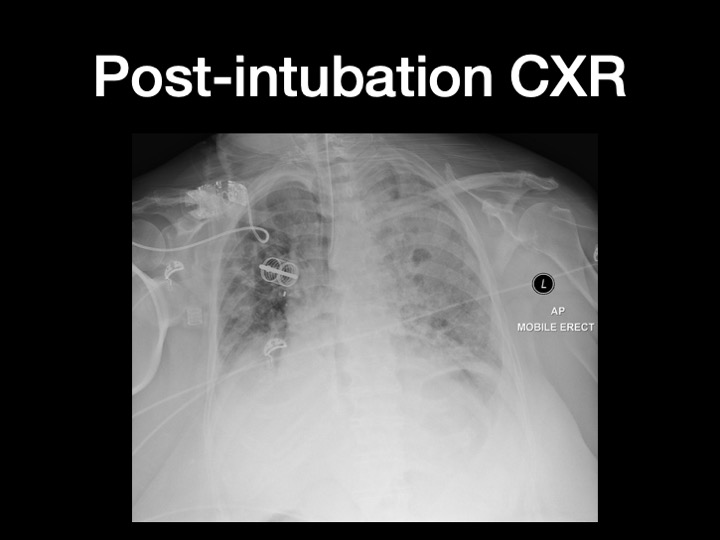

Supplement: Supplementary file 3 [file jetem-5-2-s1-supp2.jpg]

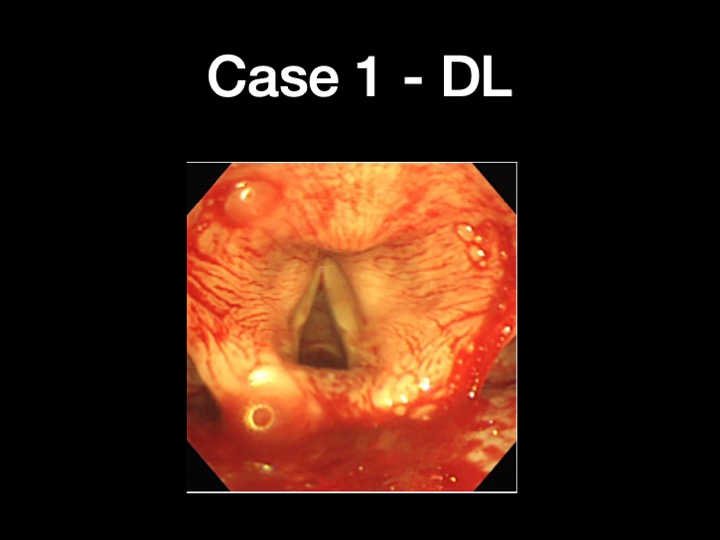

Supplement: Supplementary file 4 [file jetem-5-2-s1-supp3.jpg]

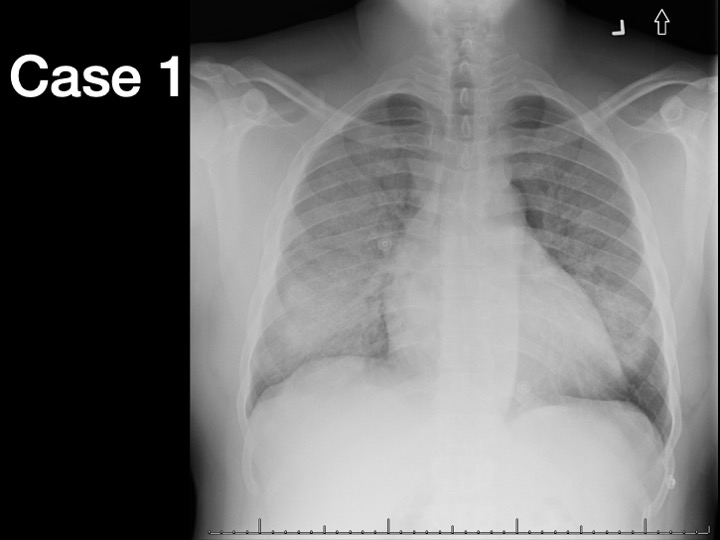

Supplement: Supplementary file 5 [file jetem-5-2-s1-supp4.jpg]

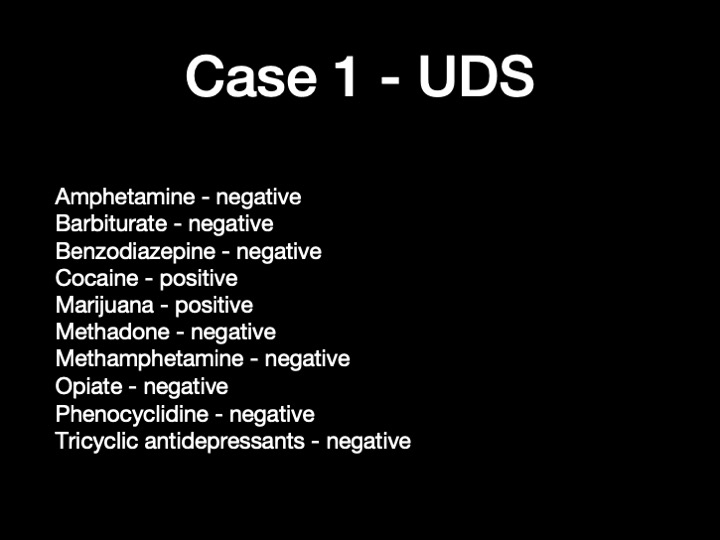

Supplement: Supplementary file 6 [file jetem-5-2-s1-supp5.jpg]

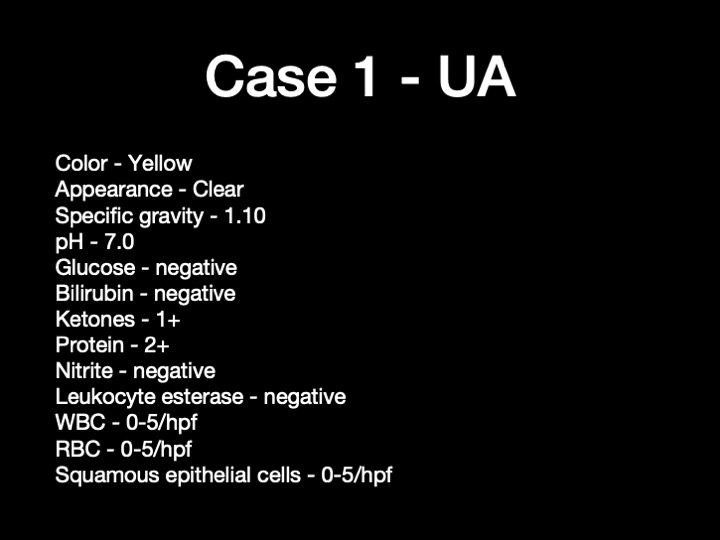

Supplement: Supplementary file 7 [file jetem-5-2-s1-supp6.jpg]

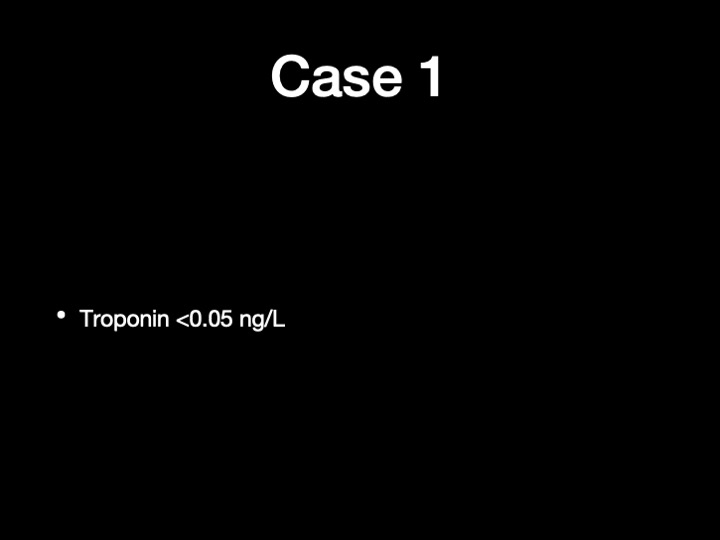

Supplement: Supplementary file 8 [file jetem-5-2-s1-supp7.jpg]

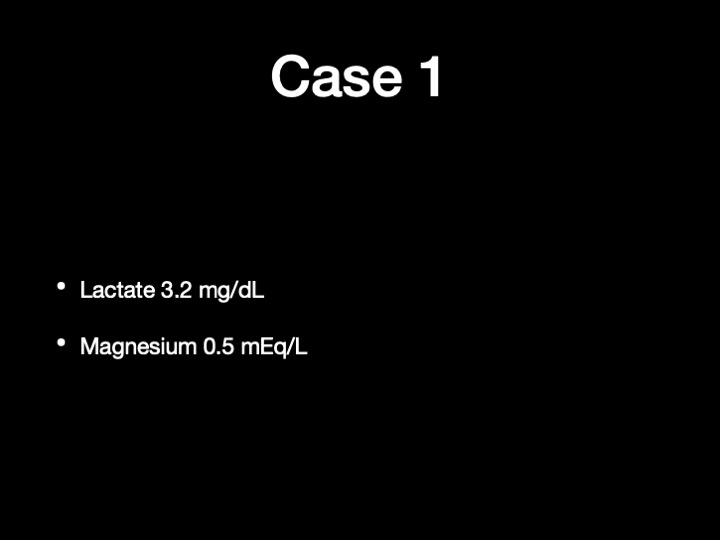

Supplement: Supplementary file 9 [file jetem-5-2-s1-supp8.jpg]

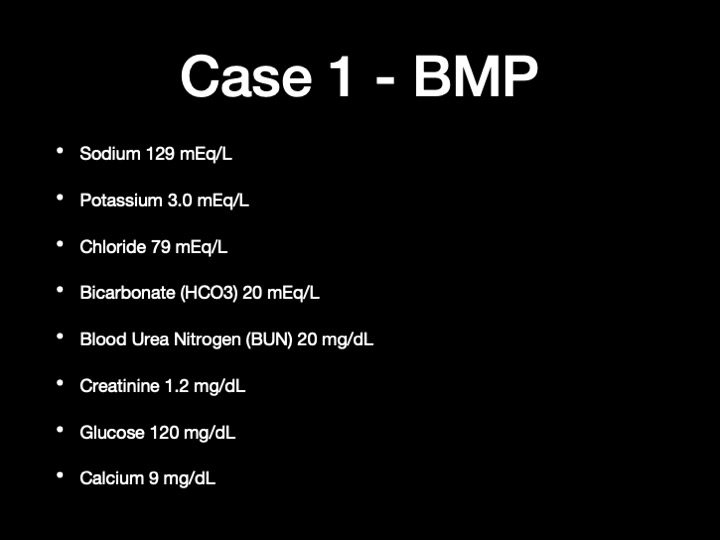

Supplement: Supplementary file 10 [file jetem-5-2-s1-supp9.jpg]

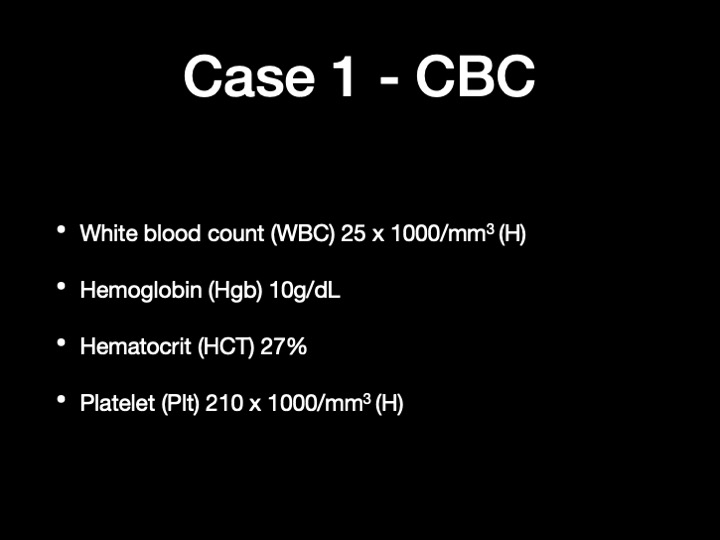

Supplement: Supplementary file 11 [file jetem-5-2-s1-supp10.jpg]

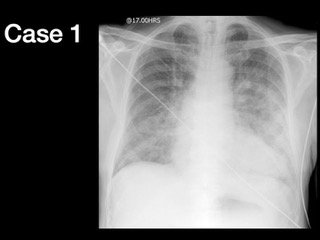

Supplement: Supplementary file 12 [file jetem-5-2-s1-supp11.jpeg]

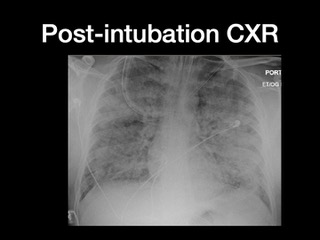

Supplement: Supplementary file 13 [file jetem-5-2-s1-supp12.jpeg]

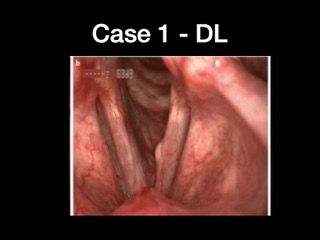

Supplement: Supplementary file 14 [file jetem-5-2-s1-supp13.jpeg]
