# Supplementary material for: Hemoptysis Due to Diffuse Alveolar Hemorrhage
Source: J Educ Teach Emerg Med. 2020 Jul 15;5(3):S1–S27. doi: 10.21980/J8ZP86 (PMC10332551; doi:10.21980/J8ZP86)
Supplement: Supplementary file 15 [file jetem-5-2-s1-supp14.pptx]

## Slide 1
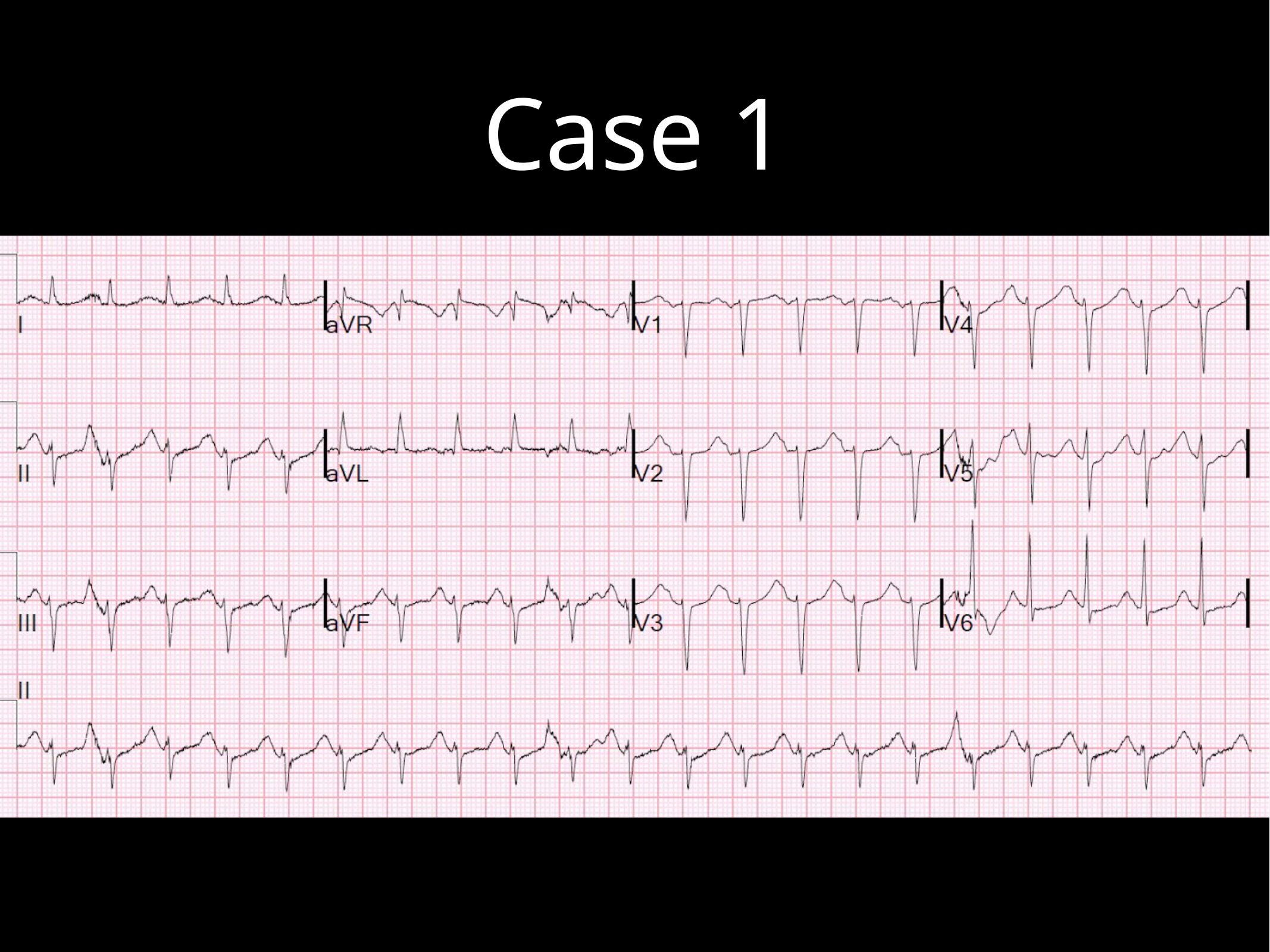

# Case 1

## Slide 2
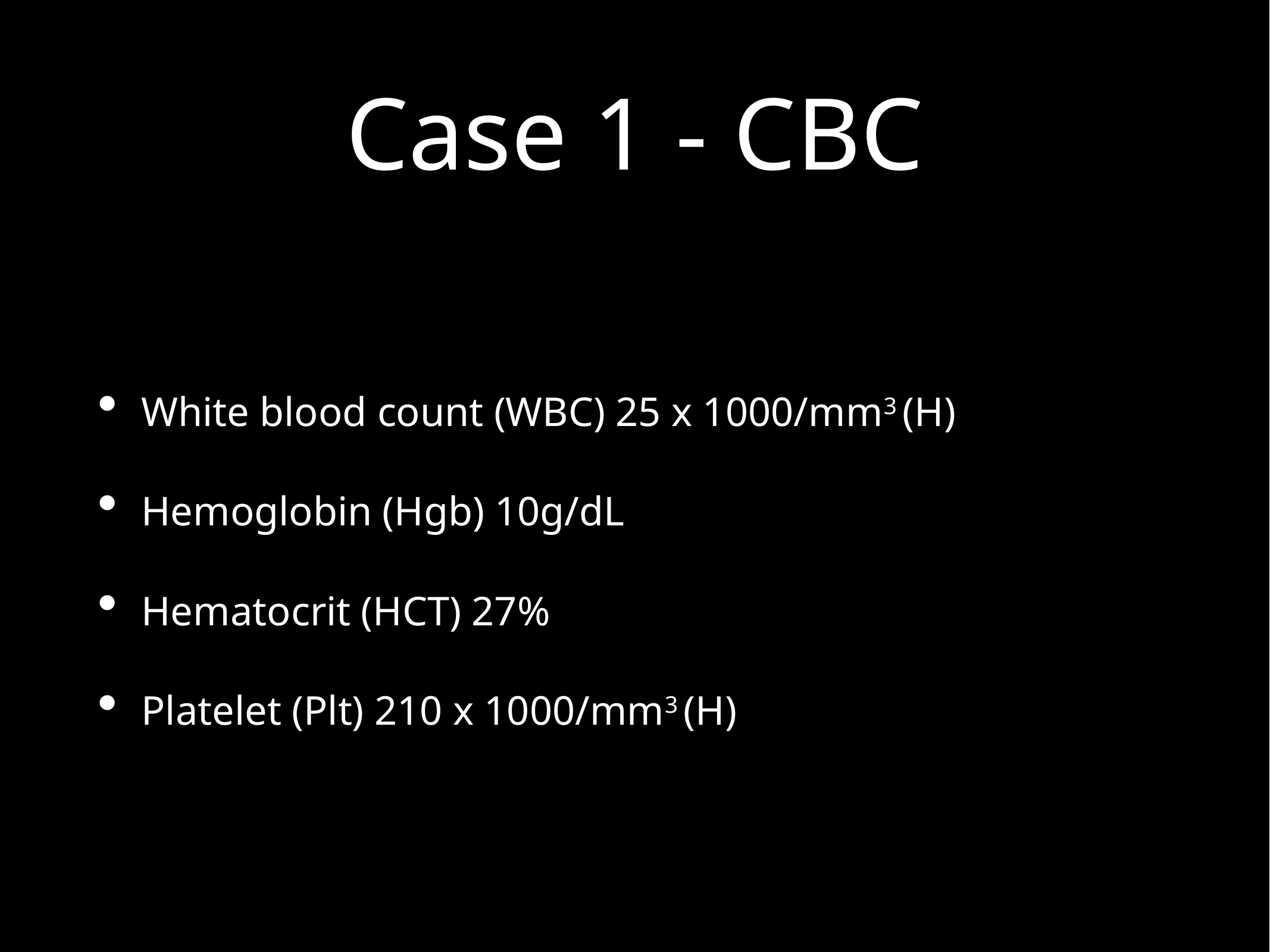

# Case 1 - CBC
White blood count (WBC) 25 x 1000/mm3 (H)
Hemoglobin (Hgb) 10g/dL
Hematocrit (HCT) 27%
Platelet (Plt) 210 x 1000/mm3 (H)

## Slide 3
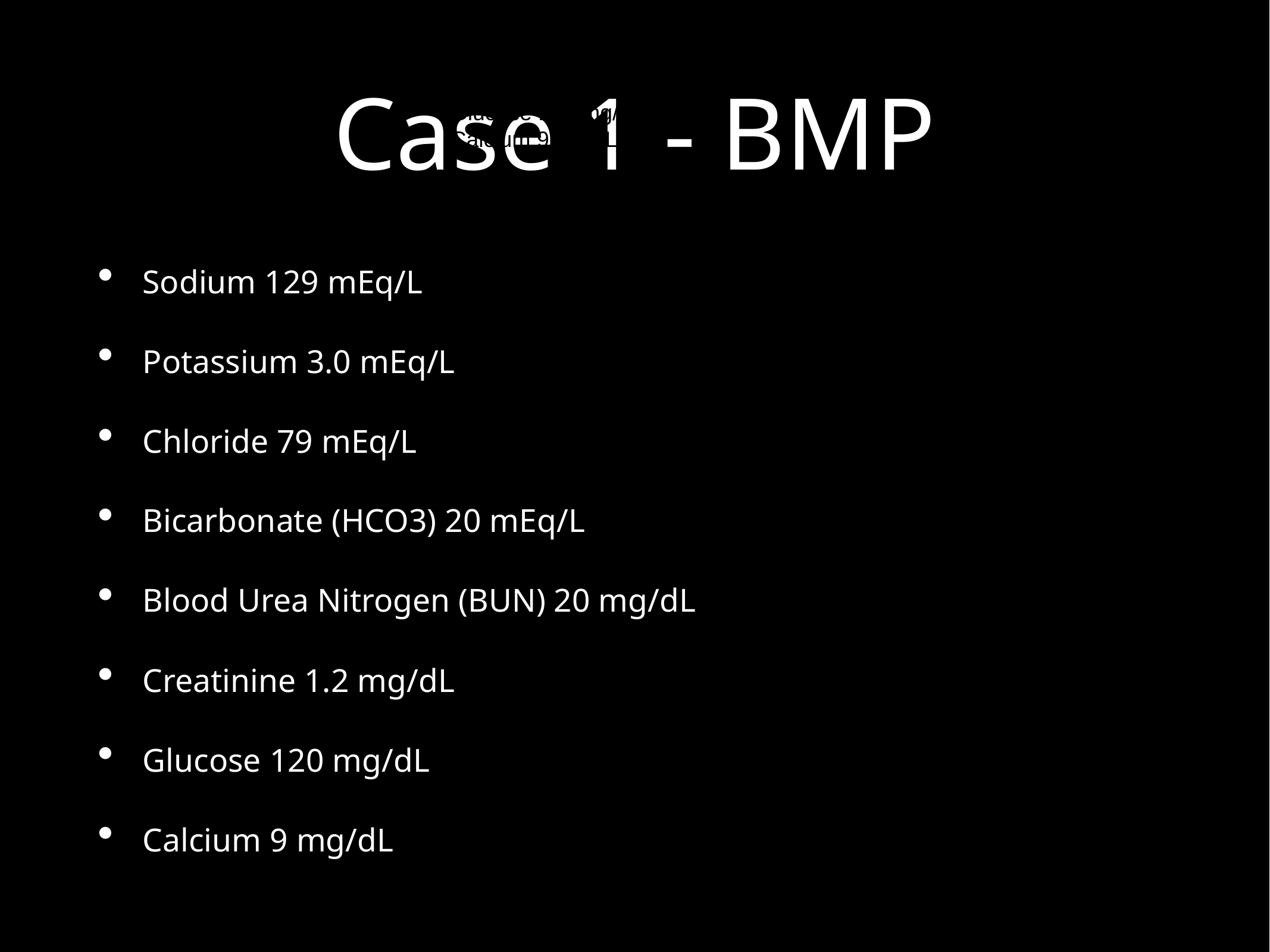

Sodium 129 mEq/L
Potassium 3.0 mEq/L
Chloride 79 mEq/L
# Case 1 - BMP
INSTRUCTOR MATERIALS
Bicarbonate (HCO3) 20 mEq/L
Blood Urea Nitrogen (BUN) 20 mg/dL
Creatinine 1.2 mg/dL
Glucose 120 mg/dL
Calcium 9 mg/dL
Sodium 129 mEq/L
Potassium 3.0 mEq/L
Chloride 79 mEq/L
Bicarbonate (HCO3) 20 mEq/L
Blood Urea Nitrogen (BUN) 20 mg/dL
Creatinine 1.2 mg/dL
Glucose 120 mg/dL
Calcium 9 mg/dL

## Slide 4
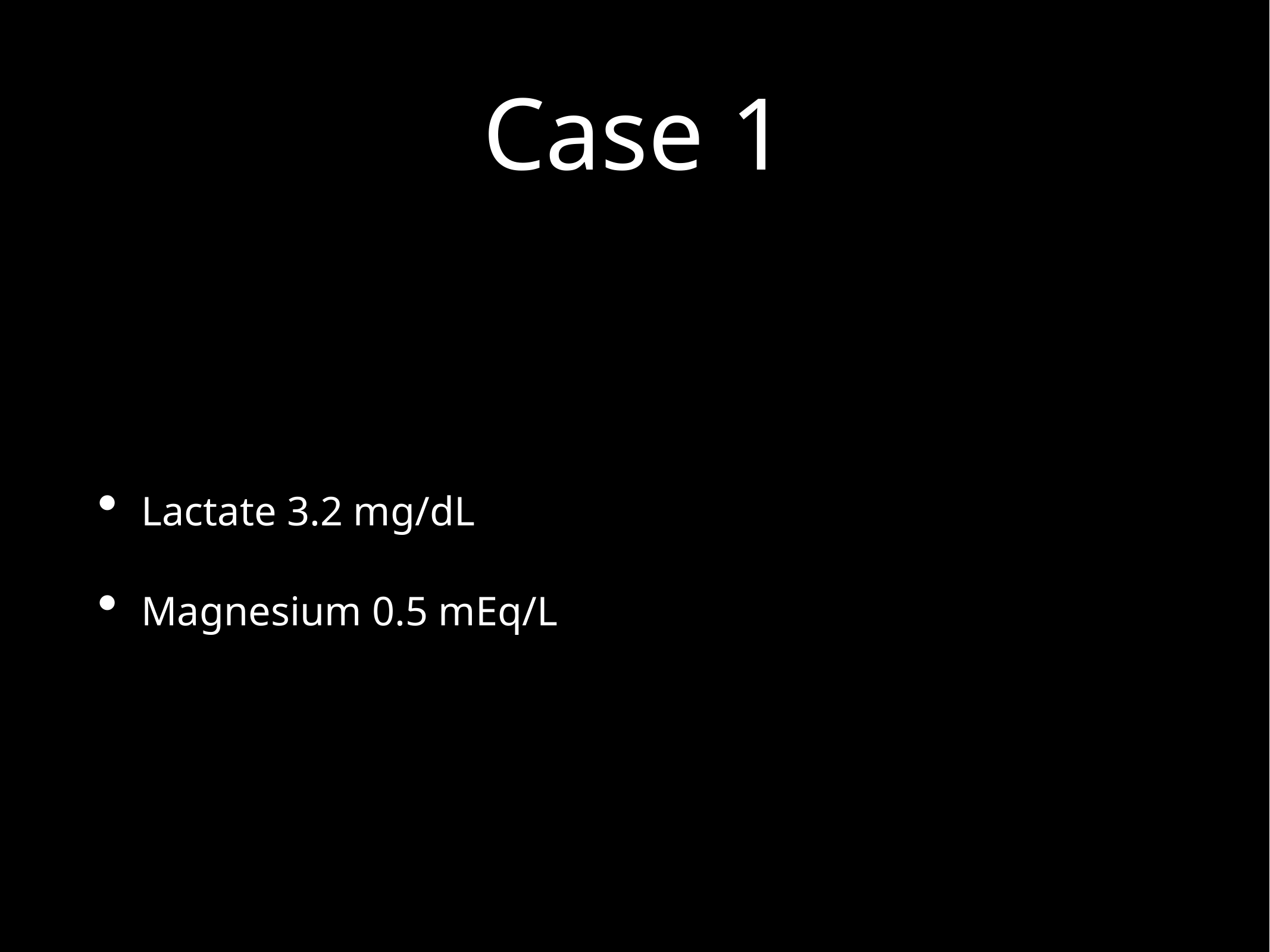

# Case 1
Lactate 3.2 mg/dL
Magnesium 0.5 mEq/L

## Slide 5
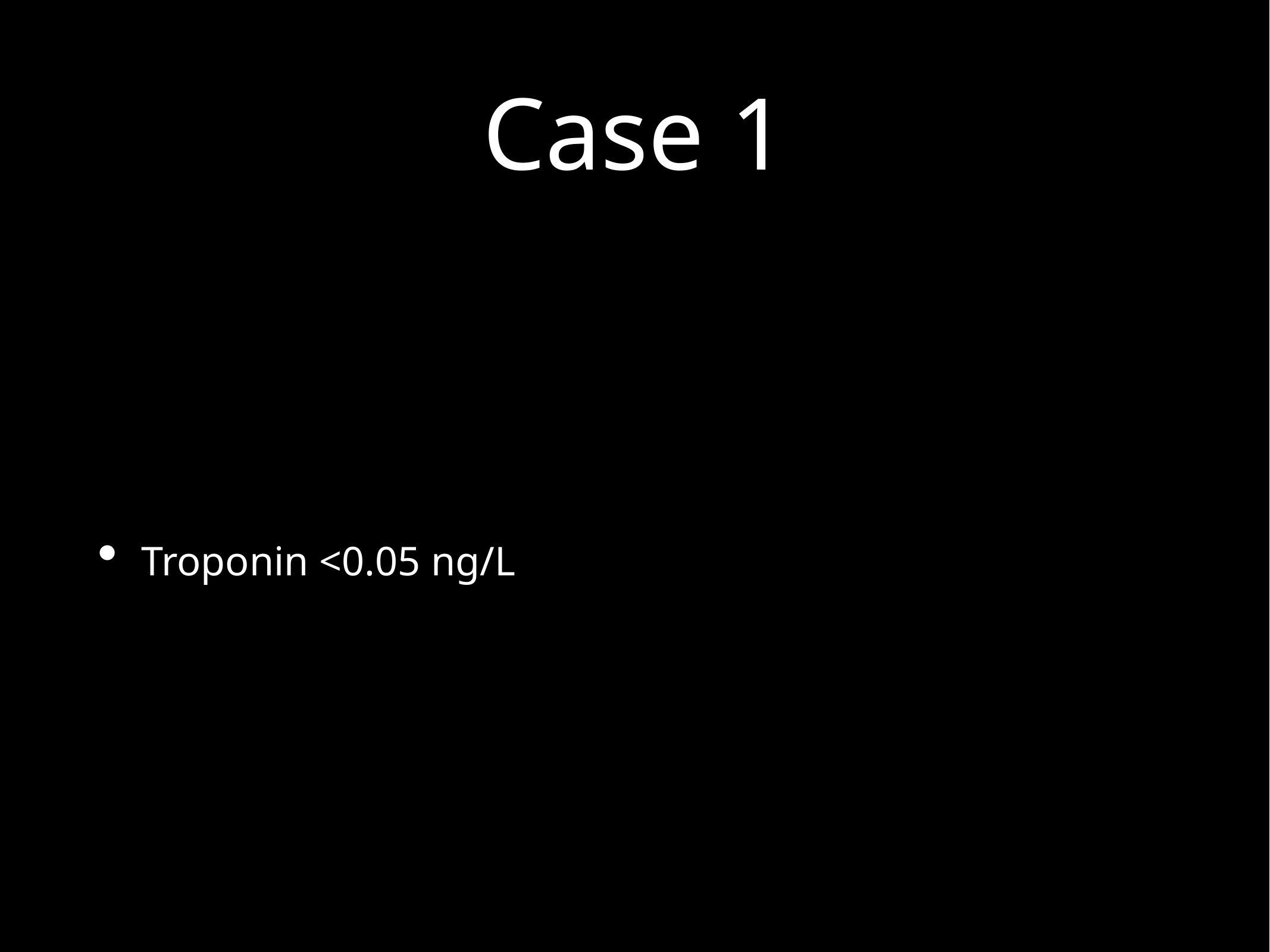

# Case 1
Troponin <0.05 ng/L

## Slide 6
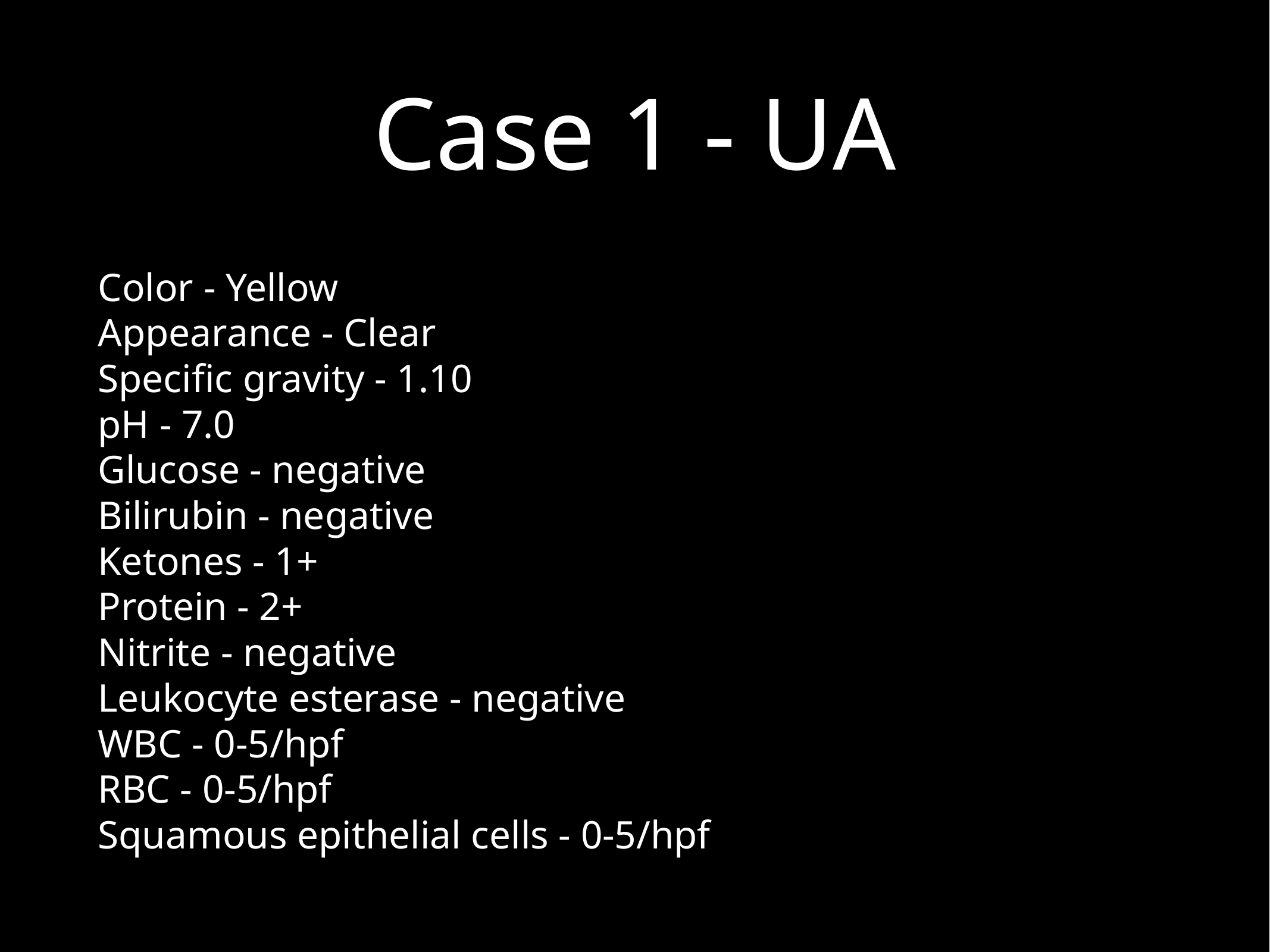

# Case 1 - UA
Color - Yellow
Appearance - Clear
Specific gravity - 1.10
pH - 7.0
Glucose - negative
Bilirubin - negative
Ketones - 1+
Protein - 2+
Nitrite - negative
Leukocyte esterase - negative
WBC - 0-5/hpf
RBC - 0-5/hpf
Squamous epithelial cells - 0-5/hpf

## Slide 7
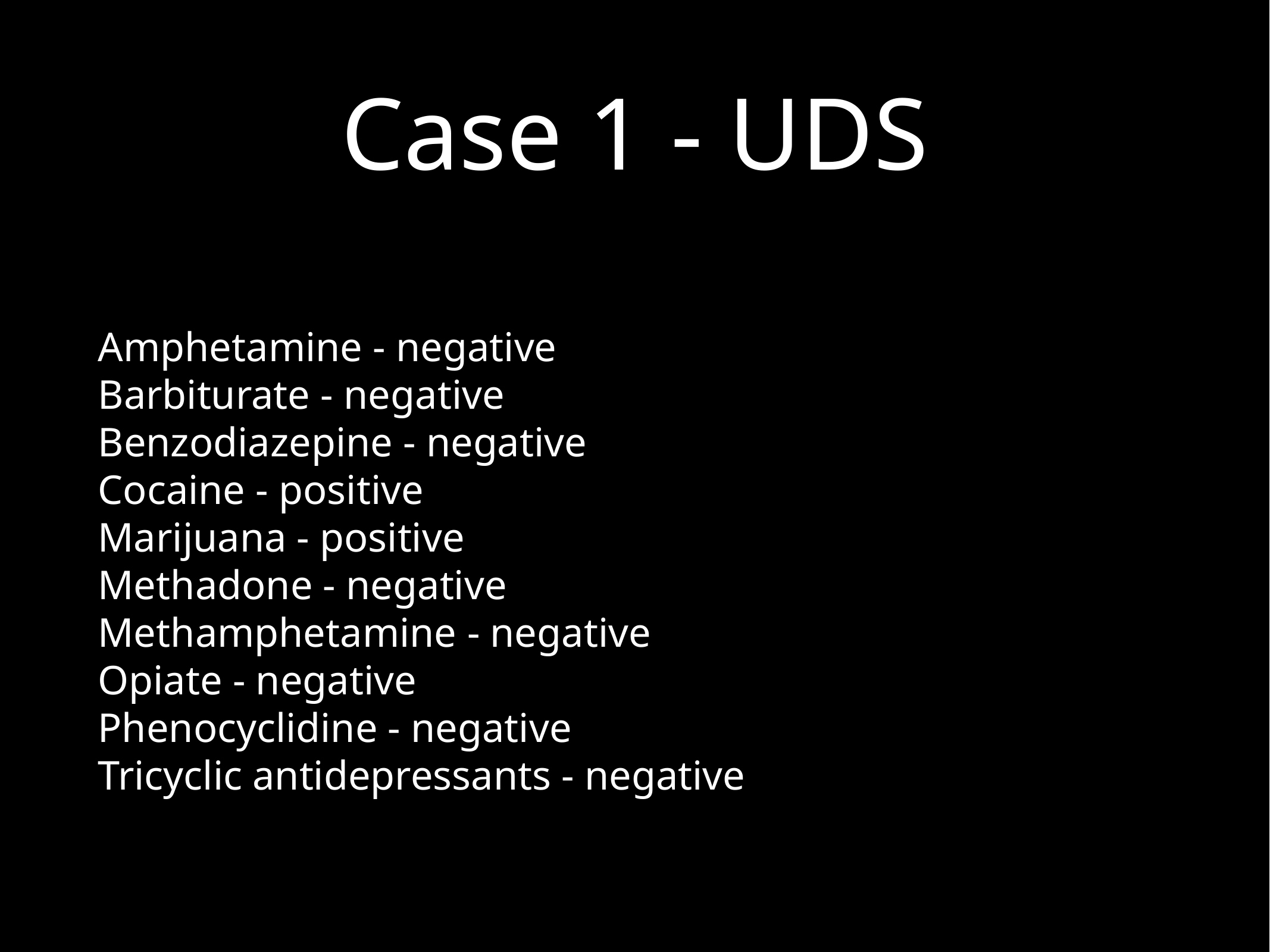

# Case 1 - UDS
Amphetamine - negative
Barbiturate - negative
Benzodiazepine - negative
Cocaine - positive
Marijuana - positive
Methadone - negative
Methamphetamine - negative
Opiate - negative
Phenocyclidine - negative
Tricyclic antidepressants - negative

## Slide 8
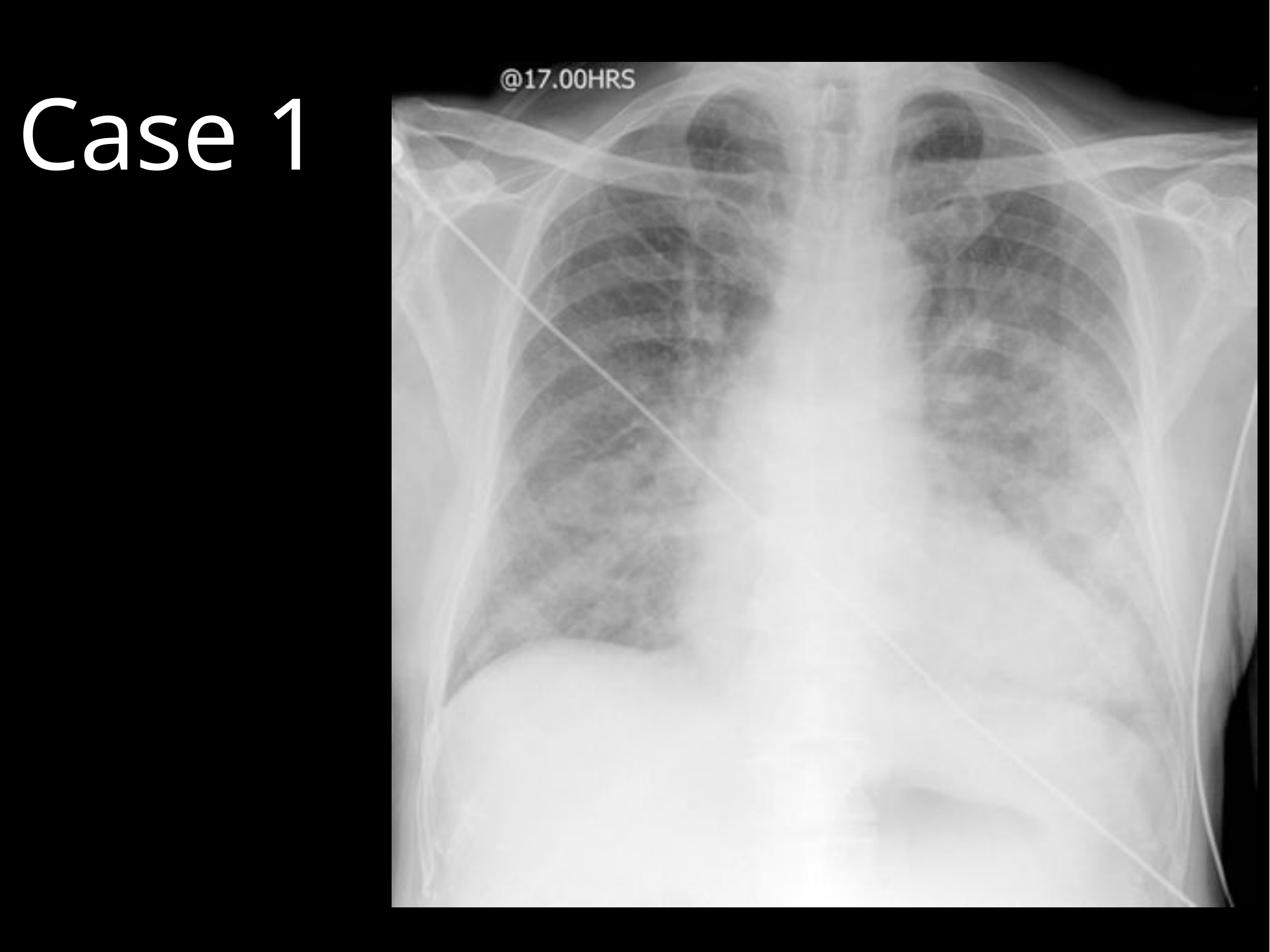

# Case 1

## Slide 9
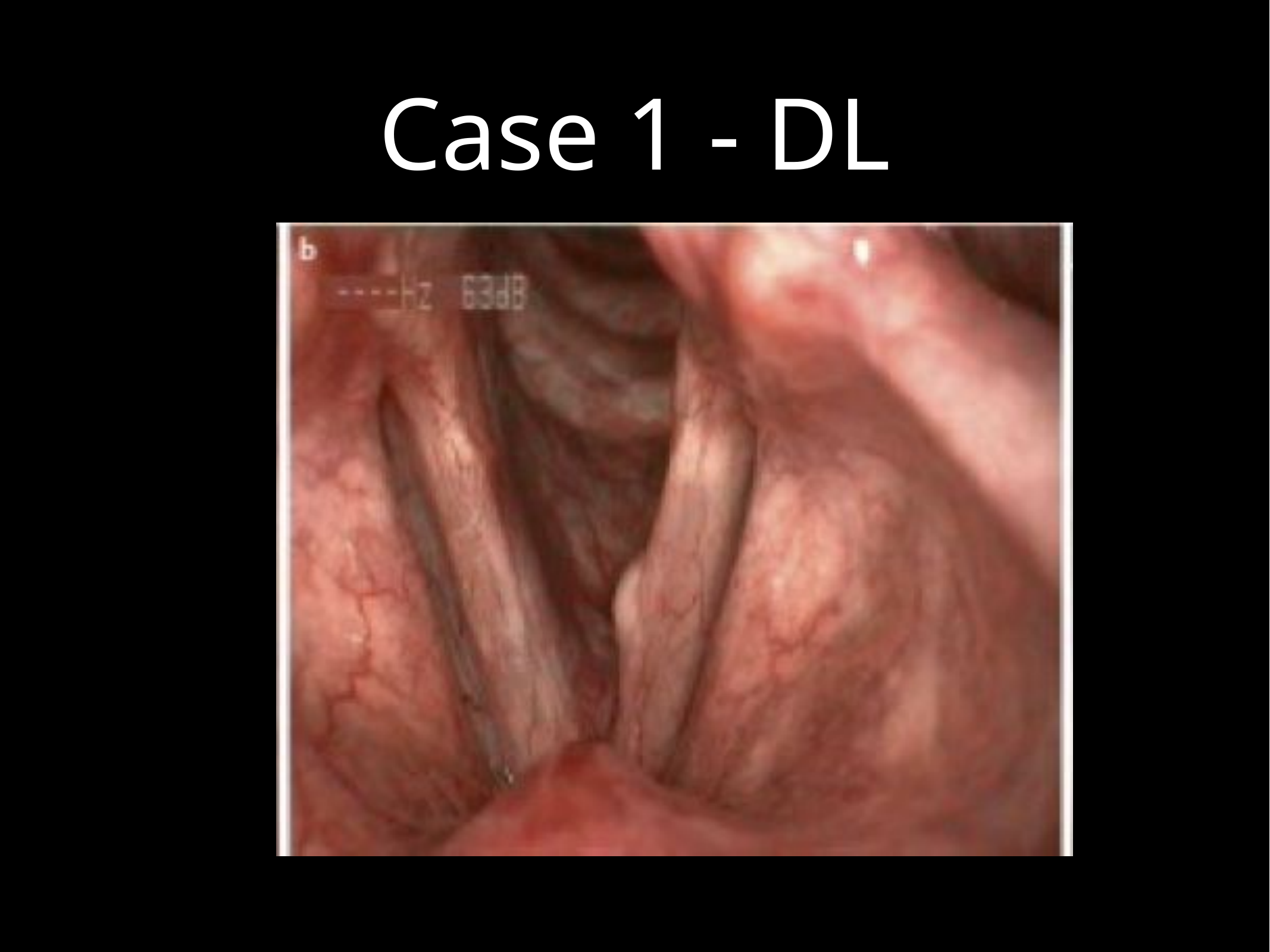

# Case 1 - DL

## Slide 10
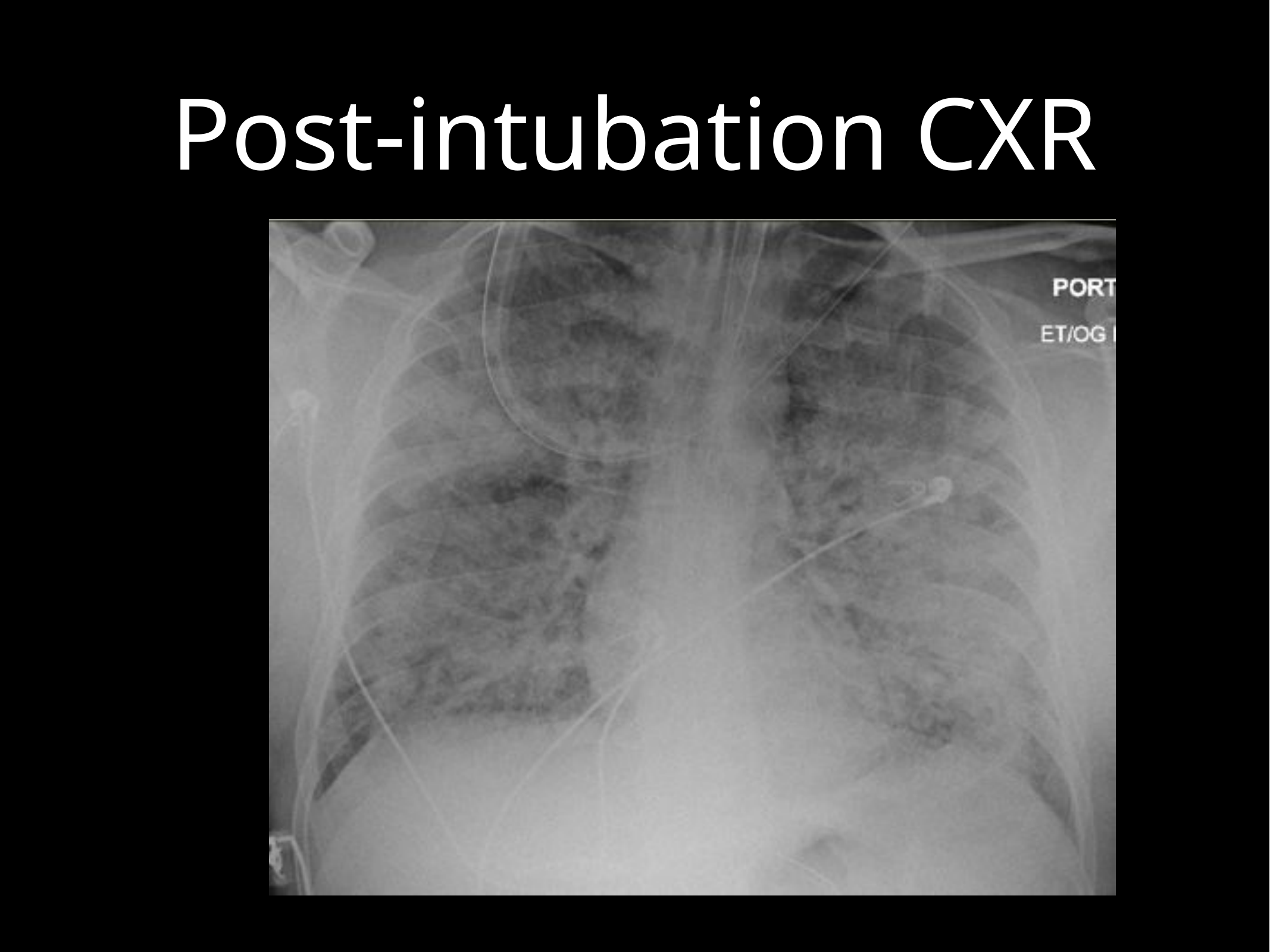

# Post-intubation CXR

## Slide 11
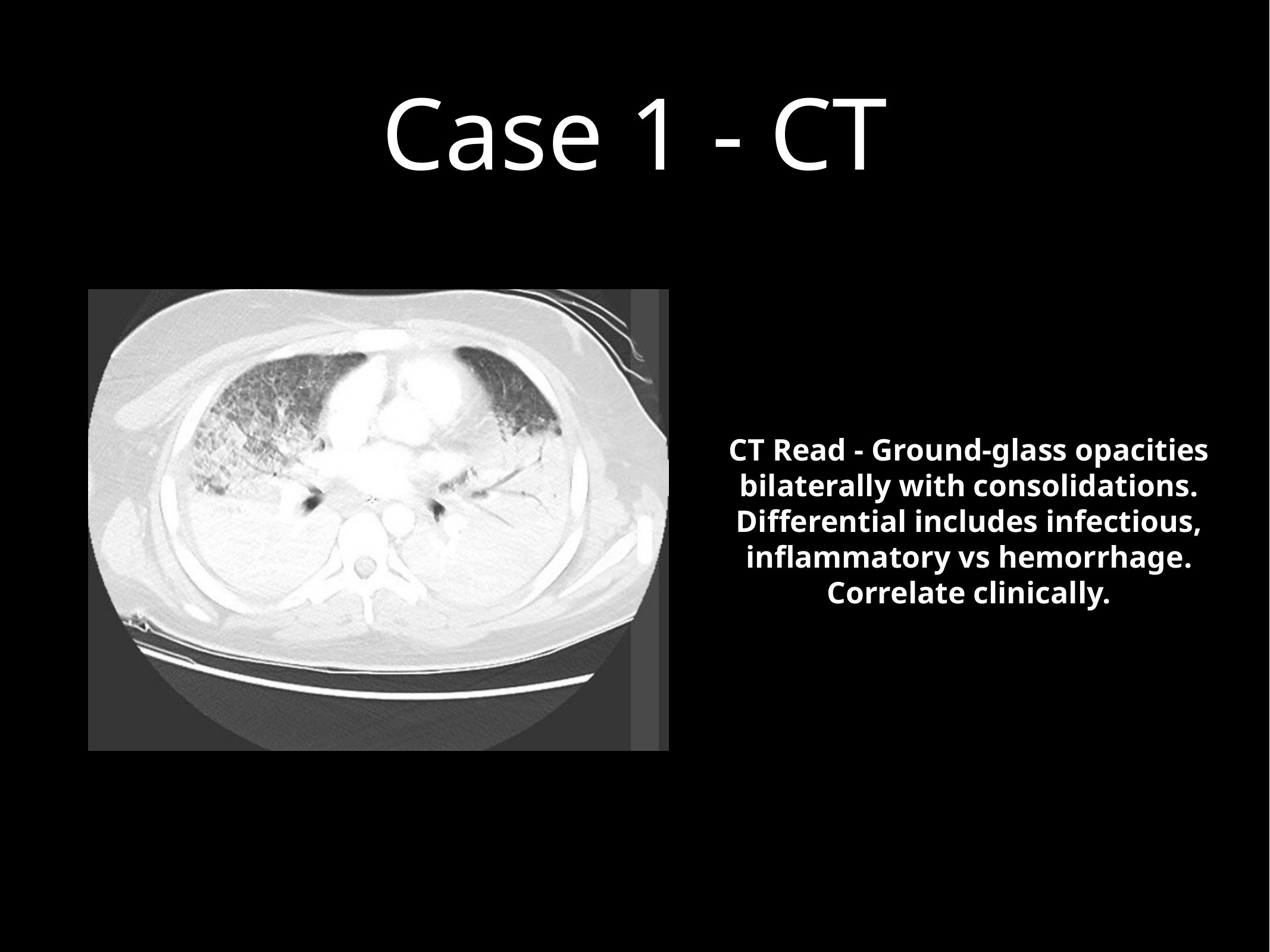

# Case 1 - CT
CT Read - Ground-glass opacities bilaterally with consolidations. Differential includes infectious, inflammatory vs hemorrhage. Correlate clinically.
